# How to write that paper

**DOI:** 10.1093/jhps/hnaa010

**Published:** 2020-04-15

**Authors:** Richard Villar

**Affiliations:** Editor-in-Chief, Journal of Hip Preservation Surgery

From time to time an author will write to ask if *JHPS* would consider a paper on a specific topic. I rarely know if this is a request out of genuine interest, or whether I am being approached to warm me up and improve a paper’s chances of acceptance. The fact is that *JHPS* will consider any paper, as long as it is related to the field of hip preservation. It does not matter if the subject has been considered previously. After all, who can say if earlier papers on the same topic were actually correct? Could yours be the paper that carries the real message?

These letters have set me thinking. How might an author improve the chances of acceptance? What are the key errors that journal editors see more or less daily? The scientific literature is filled with papers that offer advice on how to write a paper [1–6]. I have occasionally felt there are more papers about how to write than there are actually written. Naturally, I am exaggerating, but there appear to be common features in papers that may be rejected. Avoiding at least some of these mistakes can dramatically improve the chances of acceptance.

Let me start with the Title. It should not be too long, nor too short and never try to be funny. Be sure that the title matches the study you have undertaken. Bahadoran *et al*. [7] describe it well. The title of a paper, they say, ‘is like a hat on a head or the front door to a house.’ In my view, they have it in one.

Next comes the Abstract [8]. You will most likely be told the maximum number of permitted words. Never exceed this, even by one. Shorter is fine, longer is not. Simply do not go there. Edit and edit again, until you have the correct length. You would also be forgiven for focussing almost more on the abstract than the main text. The abstract is what most reviewers read from outset and is what will appear online, in perpetuity. Be sure to have suitable key words in the abstract so that your paper will be among the first to appear when a future author wishes to make a citation. Remember Google and SEO? That is how you handle your abstract. You want to be on that first page, whether it be Google or PubMed. If you need advice on key words, have a look at MeSH online [9].

Your paper then starts with the Introduction [10, 11]. Make this interesting. You are not only reporting a scientific study, you are telling a scientific story in an academic way. Readers and reviewers are human beings and respond to the same stimuli as the rest of the world. Do not send them to sleep even before they have begun. The Introduction must be relevant—keep on topic throughout—and it must carry a clear hypothesis. It is in the Introduction that your references begin to appear, in order to support your ideas. Finish the Introduction in a way that leads naturally into the next section, as one flows into the other.

The next section is Materials and Methods [12]. This is where the real meat starts. Remember that if your Abstract and Introduction are poor, the reviewer may not even make it as far as this section. Have enough information here to allow a reader to repeat your study if they wish. Be sure that you have consent from the appropriate body, including any patients, to undertake the research, and also to publish your results. If there are guidelines to follow, then follow them [13–15]. Be sure, too, that your statistics are up to speed and have no hesitation in asking a statistician to be involved, preferably before you start your study, if a study is what you have done.

Results [16] come next and this section is for what it says—results. This is not the place for discussion. Be sure to avoid repeating yourself. For example, if you say something in the text, do you need to repeat it in a graph or table? Be sure, too, that any photographs you use are well annotated and show what they are meant to display. I have doubts about the value of bar charts. There are few bar charts that cannot be more simply summarized in the text. Just because you happen to be pleased by a photograph does not mean it has to be used. Stick to your message and keep it simple. Use as few images as you can.

The Discussion [17] is not an opportunity to show off. Again, stick to message and discuss what you have found, what others might have found, and bring in more references, too. This is not the place to simply repeat your Introduction. Do not overinterpret your findings or ascribe greater significance to them than they deserve. This is also the section to own up. If you feel your study has limitations, then say so. All studies have limitations. These do not mean your work will be rejected unless a limitation is actually a fatal flaw.

Then comes the Conclusion [18]. It may feature at the very end of the Discussion, or it may be in a section of its own. Keep it short and clear. It is not an opportunity to repeat what has been said earlier.

Acknowledgements [19] may then follow. Be certain to explain why an individual is being acknowledged. What did they actually do to help the paper? Be certain, too, that anyone you have acknowledged knows they have been acknowledged. They may prefer not to feature.

References are at the end and are important; as it is to these that so many readers will go. Think hard about the sources you have cited. Does the journal to which you are submitting your paper actually feature in your list of references? If not, think again, as perhaps you are submitting to the wrong journal. I am constantly astonished how many papers are submitted to *JHPS* without the journal being cited even once. If you are going to cite a reference, be sure to have read it. At the very least be certain to read the Abstract. Read also the journal’s author guidelines. If they say no more than 25 references, than 25 is your maximum number. Please do what they say.

When it comes to format and presentation, the journal should spell out what to do. Be certain to follow the guidelines to the letter. Also be sure to use fantastic English. If this language is not your native tongue, unless you are utterly fluent, think about using an agency to top and tail your paper. At the very least, pass your submission by a native English speaker.

Do not be surprised if a reviewer accuses you of omitting some key finding from your paper when you know you actually included it.

‘Look!’ you might protest. ‘I mentioned it in line 34 of page 7. Can’t you read?’

The fact is, you may well have said something that was missed. However, if it was missed, was it because the reviewer was being dozy, or was it because your writing was not up to par? Modern writers for the mass market will often say that if an article is rejected, look in the mirror. The fault is likely yours.

So much for how you might do it. Now for what we have done. In our last issue of *JHPS*, as with all other issues, I enjoyed every last title. Several stood out, however, including the paper by Cychosz *et al*. [20] on the validation of a novel hip arthroscopy simulator. Their use of the ArthroS hip simulator showed good construct validity, and performance correlated well with the total number of arthroscopic cases reported during training. In our highly litigious orthopaedic world, I found this to be most useful.

I also enjoyed the paper by Ortiz-Declet *et al*. [21], who presented a new, dynamic clinical examination for the detection of gluteus medius tears. They established that their so-called resisted internal rotation test helped them identify gluteus medius pathology. This is most helpful in an era when gluteus medius is being so widely repaired.

And as for this issue, number 7.1, once again I find it to be filled with excellence. It is impossible to know where to begin. Nevertheless I was struck by the paper from Harris *et al*. [22], who found that experienced hip arthroscopic surgeons appear to agree when it comes to the reliability of the measurement of hip range of motion. At least there is something on which we agree.

I was also taken by the paper from Bræmer *et al*. [23], who undertook a follow-up study of 74 patients 2 years after a reverse periacetabular osteotomy. Although there was a loss to follow-up of 23%, decreased pain was associated with improved hip function and the majority of patients was satisfied with the results of their procedure. This was excellent news.

So, as ever, please enjoy this issue of *JHPS*. It is published for you, the hip preservation practitioner, and is filled from cover to cover with brilliance. I commend this issue to you in its entirety.

My very best wishes to you all.
